# Feed Safety and the Development of Poultry Intestinal Microbiota

**DOI:** 10.3390/ani12202890

**Published:** 2022-10-21

**Authors:** Dragana Stanley, Yadav Sharma Bajagai

**Affiliations:** Institute for Future Farming Systems, Central Queensland University, Rockhampton, QLD 4702, Australia

**Keywords:** nutrition, gut colonisation, microbiota, feed contamination, pathogens

## Abstract

**Simple Summary:**

Intensive gut colonisation of animals starts immediately after birth or hatch. Oral route of colonisation, and consequently the first feed, plays a significant role in the continual defining of the intestinal microbial community. The feed can influence colonisation in two ways: providing the microbial inoculum and providing the nutritional requirements that suit a specific type of microbes. In combination with environmental factors, feed shapes animal’s future health and performance from the first day of life. The objective of this review was to investigate feed safety aspects of animal nutrition from the gut colonisation aspect.

**Abstract:**

The first feed offered to young chicks is likely the most important meal in their life. The complex gut colonisation process is determined with early exposure and during the first days of life before the microbial community is formed. Therefore, providing access to high-quality feed and an environment enriched in the beneficial and deprived of pathogenic microorganisms during this period is critical. Feed often carries a complex microbial community that can contain major poultry pathogens and a range of chemical contaminants such as heavy metals, mycotoxins, pesticides and herbicides, which, although present in minute amounts, can have a profound effect on the development of the microbial community and have a permanent effect on bird’s overall health and performance. The magnitude of their interference with gut colonisation in livestock is yet to be determined. Here, we present the animal feed quality issues that can significantly influence the microbial community development, thus severely affecting the bird’s health and performance.

## 1. Background

Immediately after birth, or hatch in birds, the initial inoculum shapes the gut microbiota for life. The first bacteria to settle in the intestine can attach to epithelial cells with no competition, rapidly establish, grow, and set the intestinal environment to suit their requirements [1,2]. The first bacterial settlers have the most substantial influence on developing the host’s immune system and overall ability to thrive [1,2]. While gut microbial communities take around two years to mature in humans, the timeframe to maturity is significantly reduced in chickens. Studies report that chicken microbiota largely stabilises by day three [3]. The maturity of gut microbiota assumes the ability to resist change to a certain level.

Studies on humans report that any early adversities, from mild, such as nutritional imbalance, to major, like antibiotic administration, before establishing a mature intestinal microflora can leave permanent consequences that lead to obesity, asthma, allergic diseases and diabetes [4,5]. “One Health” relationships between human, animal and environmental microbiomes suggest that they interact and respond to challenges in a highly similar and predictable way [6], and the importance of early chick microbiota exposure has also been established [7,8,9]. 

Poultry research invested decades in optimising bird nutrition to achieve maximum health and performance. The early nutritional needs of hatchlings are well defined. However, advances in molecular microbiology and microbiota research have shed new light on the role of early chick feed, not just in providing nutrition to the host but also in providing nutrition to beneficial microorganisms and restricting the essential nutrients to pathogenic microorganisms in the first days post-hatch. This way, the early feed can contribute to forming a balanced gut microbial community. While there are many factors influencing gut microbiota colonisation and maturation, such as maternal immunity and particularly yolk sack IgY levels [10], metabolic crosstalk between the host and microbiota [11], colonisation resistance [12], breed and genetics [13], sex [14], access and bird preference to free range [15] and other host and environmental factors, this review will concentrate on the concerns with feed quality and safety issues and its possible role in gut colonisation. 

## 2. Gut Colonisation

Intestinal colonisation with the microbial community is one of the most critical events in the life of any animal, especially for the life of poultry that is born into high production stresses, high pathogen load and intensive production systems. The abundance of information on early gut colonisation comes from human research, where we can observe a highly regulated process of inoculation during natural birth, followed by boosting with breast milk. However, when contact with maternal microbiota is interrupted by an unnatural process such as a C-section and bottle feeding, the consequences to the infant’s health can be long-reaching [16,17]. Adding probiotics into the baby formula immediately post-birth in preterm babies leads to a healthier microbial community with reduced pathogen load compared to the non-supplemented formula [16]. As the microbial community matures, it assumes a more stable state capable of better resisting major pathogenic challenges. Considering the similarities in intestinal physiology between the animal species, these findings are highly relevant to the colonisation of poultry.

The situation has far more deviated from the natural gut colonisation process in industrially grown poultry. The eggs are removed from maternal influence and hatched in a clean environment deprived of natural maternal beneficial inoculum, resulting in aberrant, randomly colonised microbiota of very high batch-to-batch variability [7]. Wilkinson et al. [18] demonstrated that the lack of maternal microbiota exposure during the first week of bird’s life, achieved by using immaculately clean conditions, could not be compensated with subsequent co-housing with naturally colonised birds; once developed, abnormal microbiota entirely prevented colonisation with beneficial bacteria. After the first-week post-hatch deprivation of maternal microbiota in clean conditions, the birds were colonised with random and unusual environmental bacteria and, after weeks of co-housing with control birds, could not secure a single *Lactobacillus* species. In such disinfected and poultry microbiota-deprived conditions, inoculating the birds with *Lactobacillus agilis* post-hatch prevented aberrant gut physiology, but it led to nearly complete *Lactobacillus* intestinal dominance by a single inoculated strain which had a week of colonisation advantage with very little competition [18]. 

Thofner et al. [9] concluded that pre-hatch application by the spray of probiotics on the eggshell could be used for the colonisation of the chicken gut. In contrast, others [19] concluded that the effects of spraying cecal microbiota on the eggshells are primarily overwhelmed by the natural microbiota acquisition processes or that the eggshell microbiota is not an efficient way to colonise poultry intestine [20]. Indeed, not all probiotics provided early in life can colonise the gut, but their administration has a prolonged effect on the microbial community by stimulating the other beneficial genera [8]. Although layer chicken microbiota partially stabilises within a week, significant stresses like transporting the flock from raring to production sheds can introduce significant and permanent community alterations [21]. This indicates the complexity of poultry gut colonisation and maturation. The critical process of gut colonisation in birds’ absence of maternal microbial inoculum occurs via bacteria from the feed natural microbiota and bacteria from the environment, including shed and open range [22].

**Figure 1:**
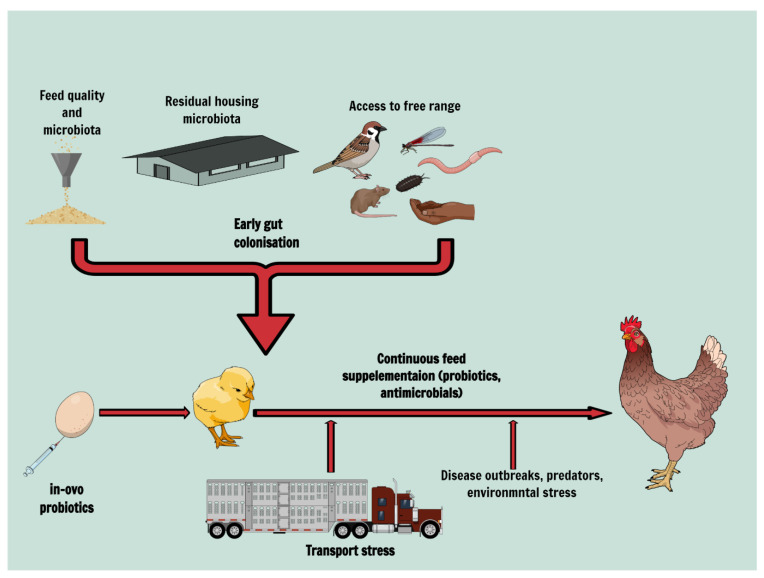
Range of factors influencing microbiota colonisation and maturation. Image created using Mindthegraph.

The influence of the environment, including shed, feed, insects, dust or exposure to the free range, on the intestinal colonisation of poultry was highly investigated in studies aiming to identify the sources of poultry pathogen colonisation and possible control. For example, the colonisation routes of *Campylobacter* [23,24], *Salmonella* [25,26], or *Escherichia* [27] are well described in poultry, and they represent the basis of on-farm biosecurity (Figure 1). Early exposure to pathogens can have long-term consequences, while colonisation with commensal microbiota can result in pathogen colonisation resistance [28]. However, avoiding premature exposure to pathogens is not easy in the poultry industry, where both the beneficial bacteria and the most aggressive poultry pathogens can arrive on the farm via a number of routes, including animal feed, as summarised in Figure 1.

## 3. Biological Contaminants in Feed

### 3.1. Microbial Contamination of Feed

One of the most critical requirements for early post-hatch feed is biosecurity because providing early pathogen access to the naïve gut could lead to mortality, lifelong colonisation, and permanent pathogen shedding. It is well established that feed can get contaminated with biological pollutants at any production stage. *Salmonella*, *Campylobacter*, *Clostridium perfringens,* and *Escherichia coli* feed contamination are at the centre of feed safety research in poultry and other livestock. In addition to bacterial pathogens, the feed can also carry antimicrobial resistance (within bacteria in feed), devastating viruses [29] or mycotoxin-producing mycobiota [30,31].

It is unreasonable to expect that the raw ingredients of the animal feed would be sterile. Finished feed pelleting turns the mashed feed into dense pellets, thus decreasing dust, food waste and reducing but not removing microbes from the feed. The process involves the application of steam and pressure similar to, for example, a basic sterilising process in an autoclave, but in order to preserve nutrients intact, the duration of steam treatment is much shorter (ranging from seconds to ~4 min total heating time), and the temperature is far less severe (65–78 °C) at 241–276 kPa recommended pressure. Pressure can be quite variable from 138 to 552 kPa) [32] compared to the sterilisation process in an autoclave, which happens with saturated steam (121–132 °C) and under the pressure of 106 kPa for at least 30 to 45 min, starting from the moment the whole batch reaches minimum 121 °C. The time of exposure increases with batch size. 

Thus, the pelleting process is disinfection and not microbiological sterilisation of the material, and it does reduce but does not remove all of the microbial load from the finished feed. Whether the remaining microbes will grow inside the feed bags depends on the transport, storage conditions and remaining moisture. Nevertheless, a range of serious pathogens is still recovered from the finished feed. Higher temperatures used in pelleting could damage the nutrients but still do not guarantee the feed would be pathogen-free, and the sanitation effects of pelleting at higher temperatures are lost during dust contamination once the bag of feed is opened and handled on the farm [32]. 

### 3.2. Salmonella

Studies show that feed and feed mills are an important source of *Salmonella* contamination in the poultry industry. Shirota et al. [33] acknowledged that it is generally presumed that baby chicks bring *Salmonella* sp. to the farm, implying hatchery contaminations, while only a limited number of studies looked at the feed as a probable source of contamination. It was reported that only trace levels of *Salmonella* could lead to young chick mortality [34]. Shirota et al. [33] analysed 4418 samples of finished layer feed in Japan and found 46 *Salmonella* strains in 143 feed samples. The isolates belonged to a minimum of 32 serovars, with the most abundant *S*. *Enteritidis*, *S*. *Livingstone*, *S*. *Bareilly*, and *S*. *Derby*. The authors concluded that the contamination was often limited to the same mills, and although the source of *Salmonella* was identified, the mills were persistently contaminated, and the decontamination process was challenging.

In another study, Sauli [35] investigated data on *Salmonella* contamination of pig feed in Switzerland to conclude that the probability that finishing pig feed contains *Salmonella* ranged from 34% (no decontamination step) to 0% (with organic acids and heat treatment decontamination step). A different study from China [36] investigated the contamination of 1077 feed samples, including raw ingredients and finished feeds, collected from feed mills, farms, and feed sales between 2009 and 2012. *Salmonella* contamination ranged from 4.7% in 2009 to the lowest of 0.66% in 2011. The contamination came from animal protein material such as meat meal, meat and bone meal, feather meal, blood meal, and fish meal but was not identified in microbial protein, rapeseed, and soybean meal, and it was found in mills, farms and feed wholesale [36].

Despite the absence of *Salmonella* positive samples in Chinese soybean [36], others [37] reported frequent *Salmonella* contamination in soybean imported to Norway, mainly from South America. This study covered data from 19 years of testing, finding that 34% of samples were positive for *Salmonella*, with variations from 12–62% each year. Additionally, the dust samples from the shiploads constantly yielded *Salmonella*. This study reported 94 *Salmonella* serovars in soybeans over 19 years, including 9 of 10 top serovars isolated from clinical cases of salmonellosis. This means that mill soybean processing practice is critical when the raw feed source is continually contaminated.

The data on feed and birds’ carriage of *Salmonella* differ between the studies and countries. Shirota et al. [33] pointed out the issues with sample collection and analysis, emphasising that each feed sample tested is usually a single sample of 30–100 g taken from a batch comprising tonnes of feed, allowing for false-negative results. They suggested that better sampling methods and strategies should be introduced. In a controlled experiment with a feed mill contaminated with *Salmonella* and *E. coli*, *E. coli* was reported as less resilient and faster to die off than persistent *Salmonella* [38]. 

Gosling et al. [38] summarised the literature on wide contamination of feed mills with *Salmonella*, concluding that the ingredient intake pits were *Salmonella* hot spots extending to all stages of growing, shipping, processing, storage, and finished feed. The authors suggested the use of less toxic organic acids for decontamination of *Salmonella* and *E. coli* instead of widely used formaldehyde-based treatments. Formaldehydes used in mill decontamination end up in the feed and could affect poultry health and microbiota [39]. Residual formaldehyde in feed can prevent recontamination by *Salmonella* [39], however, safety concerns now override these benefits. Many authors investigated improved ways to remove *Salmonella* and other pathogens from feed mills.

Standard methods of disinfection of feed mill food contact surfaces were based on “sequencing” of raw feed ingredients so that those most likely to carry pathogens are left for last, followed by flushing of equipment with the pulse of animal food such as chemical treated rice hull [40] to clean the equipment and minimise leftover pathogens. The critical issue was the breaking of biofilms formed on the mill equipment. Muckey et al. [41] investigated methods of sanitation following controlled contamination with *Salmonella* using a commercially available essential oil blend or rice hulls treated with medium-chain fatty acids finding that both treatments can reduce the contamination compared to control. The authors suggested that feed “sequencing” can reduce *Salmonella* contamination on manufacturing surfaces, particularly when flushing is combined with chemical treatments.

### 3.3. Campylobacter

*Campylobacter* is one of the leading causes of human gastroenteritis and, like *Salmonella,* it is commonly found in feed, but its’ disease burden was recently amplified with the consumer push for open and free-range poultry production due to *Campylobacter* abundance in soil and environment, including animals and insects [42]. Unlike heavily researched *Salmonella* sp., the data on *Campylobacter* in feed is limited, although it is recognised that poultry feed can be a source of *Campylobacter* colonisation [24], a fact well established in humans where food is the dominant source of *Campylobacter,* which is recognised as a foodborne pathogen [43]. *Campylobacter* colonisation in commercial chickens occurs predominantly via drinking water or feed (reviewed in [42]). Although *Campylobacter* is sensitive to food processing stresses, especially high temperatures, compared to other foodborne pathogens, it is reasonably more cold-tolerant than other pathogens. Refrigerating food supports *Campylobacter* survival on dry surfaces for a few weeks instead of a few days [42].

The growth of *Campylobacter* in the feed depends on the resident microbiota of the feed and supplements affecting feed microbiota. Richardson et al. [44] directed five experiments to investigate the recovery of *Campylobacter* from feed and the impact of feed moisture, water activity, pH, and existing microbial community. The authors inoculated *Campylobacter* in feed under various conditions and reported that *Campylobacter* was viable even ten days post-inoculation when using media supplemented with antibiotics compared to one day in unsupplemented media. The authors suggested that antibiotic supplementation was likely suppressing native microbiota in feed, thus helping *Campylobacter* persist. This study points to the importance of background feed microbiota and carefully evaluating antibacterial supplements’ efficacy against major pathogens before adding them into feed formulations. 

Iovine and Blaser [45] reviewed the role of antibiotic supplementation in the emergence of deadly antibiotic-resistant strains of *Campylobacter,* the implication of this is that being very efficient in rapidly acquiring resistance to antibiotics, *Campylobacter* will overgrow in the environment where those antibiotics are still used with the other competition suppressed. The role of the presence of other bacteria in feed was highlighted in a study that investigated the effects of fermented, *Lactobacillus*-rich feed on animal susceptibility to getting colonised with *Campylobacter* [46] to find a nine times lower probability of shedding *Campylobacter* in fermented feed group.

### 3.4. Clostridium Perfringens

Diverse toxin-producing *C. perfringens* is one of the major sources of severe intestinal disease in animals and humans. This common pathogen of poultry causes significant economic loss to the farmers. The colonisation of the birds with *C. perfringens* is an early event [47]. The contamination can spread to the farm environment and equipment like fans, fly strips, dirt at the shed entrance, and workers’ boots. The birds can also be infected with *C. perfringens* while transported in contaminated coops or boxes where young hatchlings get introduced to this pathogen. As a consequence of its ubiquity, *C. perfringens* is also recovered from broiler carcasses after refrigeration [47]. Tessari et al. [48] reported *C. perfringens* isolated in raw and finished feed, including meat meal, feather meal, organ meal, and finished feed, indicating that the feed could be the initial source bringing *C. perfringens* to the farm. Once established in the dirt, workers’ footwear, fans, and air ducts, the *C. perfringens* would persist on the farm even if the feed was pathogen-free. Others also reported that *C. perfringens* comes mainly from animal protein-rich feed ingredients like fish meal, bone meal, meat and bone meal and dry fish [49].

### 3.5. Escherichia Coli

*E. coli* is a standard member of intestinal microbiota across the species, and most of its members are not pathogenic. However, those that are pathogenic are significant causes of poultry mortality worldwide and represent a persistent and continual issue in the production of both layers and broilers. The origins of pathogenic *E. coli* in the flock can also be traced to feed contamination. Ge et al., [50] compared the presence of *Campylobacter, Salmonella, Escherichia coli*, and *Enterococcus* in feed and found that they were present in zero, 23%, 39%, and 87%, respectively, in a total of 201 feed ingredient samples. However, in low and middle-income countries, the percentage of *E. coli* poultry feed contamination can be as high as 58% [51] to 100% [52], voicing the need to increase feed quality control to prevent mortality and disease outbreaks in birds and the human population. Da Costa et al. [53] recovered 163 different *E. coli* strains from 23 samples of commercial broiler feed and 66 samples of raw feed ingredients, which indicates the diversity of the species present in feed and that the presence of *E. coli* does not always imply pathogenicity. 

### 3.6. Bacteriophages

Bacteriophages (phages) are the bridge between bacteria and viruses. As viruses that prey on bacteria, phages are the biosphere’s most abundant and ubiquitous organisms. Similar to enteric viruses, bacteriophages are a valuable indicator for modelling their fate and movement. In contrast, enteric bacteriophages, especially those infecting *Salmonella* and *E. coli,* are used for source-tracking and monitoring faecal contamination of water and the environment [54,55,56,57]. Considering that the richest sources of phages include sewage, wastewater, animal farm effluent, and other faecal content abundant materials, most of those materials, with or without processing and purification, ultimately end up in waterways and get absorbed in the soil; opening multiple routes of faecal contamination into the raw feed, of both animal and plant origin [56]. Maciorowski et al. [58] reported that the increased presence of bacteriophages in animal feed is an indicator of faecal contamination. This presents an unexploited opportunity to include phage monitoring in raw feed quality assessments. 

### 3.7. Feed Microbiota

Instead of investigating feed and raw feed ingredients using classic PCR and other methods that target specific species or genera, a recent study [22] utilised next-generation DNA amplicon sequencing from raw and finished poultry feed to find that each feed source carried a rich microbial community. Investigated raw ingredients included meat and bone meal, wheat, corn, canola, barley, soybean, millrun, sorghum, poultry oil, oats, limestone and bloodmeal from four geographically distinct feedstuff suppliers. In agreement with reported pathogen tracing to high protein raw feedstuffs, the authors established that the meat and bone meal and bloodmeal samples contained the most complex microbial community, highly distinct from one another. Unique and dissimilar microbial communities were reported in poultry oil and limestone, distinct from highly overlapping microbiota found in the grain and seed samples: barley, canola, corn, millrun, oats, sorghum, soybean meal and wheat. 

Feed microbial profile contained four phyla, in order of abundance: *Actinobacteria, Proteobacteria, Firmicutes* and *Bacteroidetes* and 50 genera that included both beneficial like *Bacillus, Bifidobacterium, Lactobacillus* and *Ruminococcus*, as well as pathogenic *Clostridium, Enterobacter, Staphylococcus* and *Streptococcus*. No *Salmonella* and *Escherichia* were detected in this study. The authors followed the feed microbiota through the intestinal sections to find that different taxa from feed likely persisted in different gut sections investigated, including the cecum, ileum and excreta. Additionally, the feed mill source of raw and finished feed had a substantial influence on microbial communities in feed, and the feed mill’s geographic location also played a role. 

Even though people used bacteria within the grain throughout history to start sourdough fermentation, there is not much literature on microbial communities in grains. Cereal grains are composed mainly of starch, and it was reasonable to expect that they would carry beneficial fibre/starch-loving probiotic bacterial strains and could be a good source of starch-degrading enzymes. It was reported that whole-grain oats carry probiotic lactic acid bacteria [59]. Carrizo et al. [60] investigated lactic acid bacterial microbiota of quinoa grains and spontaneous quinoa sourdough to isolate and identify a range of *Lactobacillus* species, including multiple strains of *L. plantarum*, *L. rhamnosus*, *L. sakei*, *Pediococcus pentosaceus*, *Leuconostoc mesenteroides*, *Enterococcus casseliflavus, E. mundtii, E. hirae, E. gallinarum, Enterococcus* sp., and *E. hermanniensis*. They continued to investigate the enzymatic and nutritional benefits of these strains to conclude that rich probiotic microbiota present in quinoa carries a rich starter culture able to increase the nutritional value of grains. 

While investigating rumen starch-hydrolysing bacteria (SHB) possessing active cell-surface-associated alpha-amylase activity, using fluorescence in situ hybridisation, Xia et al. [61] discovered that 19–23% of the total rumen bacterial cells colonised via attachment to particles of four cultivars of barley and corn used for feed. Most of these bacteria were members of the *Ruminococcaceae*. By microscopical inspection of whole and crushed corn and barley cell wash, the authors identified cocci of different sizes, single or in chains, and rods of different morphology in all samples. The proportion of barley grain in the diet greatly impacted the percentage abundance of total SHB and *Ruminococcaceae* SHB in these animals. 

Pan et al. [62] investigated the ways to reduce *Fusarium graminearum* in wheat to control *Fusarium* head blight and subsequent contamination of grain with mycotoxins. They evaluated bacterial endophytes isolated from wheat grain for antagonistic ability against *F. graminearum* under field conditions. They identified a range of grain endophytes with one isolate of *Bacillus megaterium* and three of *Bacillus subtilis,* significantly inhibiting the growth of *Fusarium* on grain. 

Endophytes are endosymbionts, most likely bacteria or fungi, that live inside or on the plant in a mutually beneficial relationship and therefore are a big part of the plant and seed residential microbiota. Bacterial and fungal endophytes are well-reviewed and documented in grains [63,64,65] and in legumes [66], thus adding more evidence to the observation of grain microbiota. The high prevalence of probiotics in grains and above discussed high prevalence of pathogens in protein-rich feedstuffs indicate that the first feed offered to hatchlings, selected and formulated to promote the growth of probiotics and inhibit pathogens, should be grain-based and rely on grains and cereals as a protein source for the first few days of gut microbiota establishment. This process needs further research and optimisation to ensure that controlled gut colonisation does not compromise performance.

## 4. Chemical Contaminants in Feed

### 4.1. Mycotoxins

Mycotoxins are secondary metabolites of filamentous fungi that are causing massive losses to agriculture worldwide. Aflatoxins, ochratoxin A, deoxynivalenol patulin, fumonisins, zearalenone, trichothecenes, fumonisins and ergot alkaloids are presently the most important in feed safety [67]. Furthermore, the range of fungal species that produce these toxins is broad, including *Fusarium, Aspergillus, Penicillium*, and *Claviceps* species. Fungi are widely distributed in nature, foods, and feedstuffs from all parts of the world, especially in tropical climates of high rain and humidity. Mycotoxins in food and feed constitute a significant issue for animal and human safety, and they are comprehensively reviewed by many [68], specifically in pig and poultry feed [69]. In a comprehensive study from humid tropical Malaysia, the authors report an abundance of mycotoxins in peanuts, cereals, cocoa, spices, feeds and nuts consumed in Malaysia [70]. Moreover, spices, oilseeds, milk, eggs, and herbal medicine products were also contaminated. Malaysian rice, oat, barley, maise meal, and wheat were contaminated with some of the most toxic mycotoxins [70]. 

Mycotoxins are carcinogenic, mutagenic, teratogenic, cytotoxic, neurotoxic, nephrotoxic, estrogenic, and immunosuppressant [67], and they affect gut microbiota most negatively by increasing the abundance of pathogens and reducing or eliminating beneficial bacteria (reviewed in [71]). Gao [72] reviewed the effects of mycotoxins on the leaky gut and intestinal barrier, including compromised intestinal integrity, thinned mucus layer and imbalance of inflammatory markers in addition to the disturbed microbial community. Others have also reviewed other targets of mycotoxins-gut mucus layer and microbiota [73]. Therefore, even traces of mycotoxins in early hatchling feed would disturb bird health and intestinal colonisation, with likely consequences for the bird’s long-term health and performance.

### 4.2. Heavy Metals

Maximum allowed concentrations of heavy metals in livestock feed are recognised as a public health concern and tightly regulated in countries worldwide. Heavy metals are the fourth most often notified hazard in Rapid Alert System for Food and Feed (RASFF) [74]. In some countries like in European Union, firm actions are taken to standardise proficiency tests for the determination of heavy metals in feed [75]. Testing for heavy metals in feed is often performed together with mycotoxin testing. Heavy metal contamination differs from country to country and depends on heavy metal pollution levels. For example, all of the tested 40 feed samples in a study from Iran [76] had acceptable Pb concentrations, while a high portion of feed samples had As, Cd and Hg above the maximum limits.

The consequences of poor testing in livestock feed can translate to human health. For example, in a study from Pakistan, Kabeer et al. [77] tested Ni, Pb, Zn, Mn, Cr, Cu and Se concentrations in poultry eggs to find that concentrations of Pb, Cr and Se in egg white, egg yolk and both feed and water were above permissible limits in tested farms and backyard birds [77]. In India, heavy metals (Cu, Zn, Cr, Pb, and Cd) originating from feed were found in milk in excessive concentrations [78]. The main concern is how heavy metal contaminated milk and eggs could affect young children whose diets are often rich in milk and eggs.

In addition to various methods developed to remove heavy metal contamination from the feed, novel approaches to this significant feed issue are desperately needed. Recently, Yang et al. [79] tested 11 maise varieties in soil experimentally polluted with Cd, As, and Pb to identify cultivars with low seed uptake of heavy metals. The hypothesis was that heavy metals might be accumulated in non-edible parts of the plant in some varieties, thus “hitting two birds with one stone” and harvesting unpolluted seeds while using the rest of the plant to decontaminate the land. Major differences between the varieties were identified, providing a new perception in dealing with soil pollution with heavy metals and pointing towards the development of improved varieties.

Due to the ability of fish to concentrate heavy metals from polluted waters, heavy metal concentrations in fishmeal are of concern [80], especially in fish collected in proximity or downstream from industrial waste disposal sites [81]. Furthermore, some heavy metals accumulate in black soldier fly [82]. Heavy metals are known for promoting antimicrobial resistance in a similar way to antibiotic addition [83,84,85] and for a range of adverse effects on the host-microbiota [86], general toxicity to microorganisms [87], plants and humans [88] and their ability to concentrate in both chicken meat [89] and plant feed products [88] calls for a cautious approach to heavy metals in feed used in early bird’s life.

### 4.3. Pesticides and Herbicides

Other common feed contaminants include pesticides and herbicides that readily accumulate in feed. A range of highly sensitive methods has been developed for screening over 100 pesticides in feed [90]. The most susceptible poultry feedstuffs include cereal such as wheat, rye, barley, oats, maise, buckwheat and others [91]. Additionally, the runoff into the waterways ensures a high presence and accumulation of both pesticides and herbicides in fish [92]. Pesticides are highly toxic and, in sufficient concentrations, fatal for humans [93], while in lower concentrations, they disrupt microbiota and cause serious health problems [94,95]. 

Glyphosate is the most highly used herbicide in agriculture, with recently identified carcinogenic effects [96]. Glyphosate is the most challenging herbicide accumulated in feedstuffs and livestock feed. The consequences of glyphosate in feed for livestock health and productivity were recently reviewed [97,98], summarising detrimental effects on animal health, including neurological damage and microbiota impairment [96]. Surprisingly, *Clostridium* and *Salmonella* are highly resistant to glyphosate resulting in an imbalance between beneficial and pathogenic microorganisms. Furthermore, glyphosate-induced clostridia overgrowth is linked to neurological toxicity [96].

### 4.4. Other Feed Contaminants 

There are many more chemical feed contaminants that include residual chemicals such as antibiotics introduced via cross-contamination and lack of equipment cleaning between the batches of feed [99,100] to radiation toxicity [101] accelerated after Chernobyl and other more recent nuclear disasters, industrial waste products [102] and estrogenic polychlorinated biphenyls [103].

**Figure 2:**
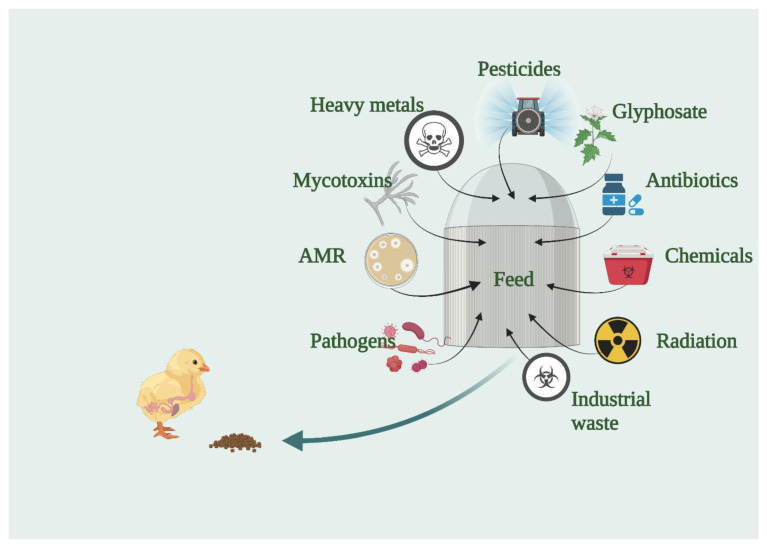
Possible feed contaminants that can disrupt early gut colonisation. Created with BioRender.

Figure 2 provides a summary of all the above-discussed feed safety issues that will likely interfere with intestinal health and early gut colonisation. The overwhelming list presented includes feed and environmental pathogens, AMR, mycotoxins, heavy metals, pesticides, herbicides (glyphosate), antibiotics, chemicals, radiation and diverse industrial waste, thus accentuating the need to identify the consequences of early exposure and the methods to mitigate their presence and negative influence on the immune and gut health and overall bird welfare.

## 5. Conclusions

Microbial colonisation of the gastrointestinal tract begins as soon as young chicks are hatched and exposed to the external environment. However, in addition to the environmental, chemical and biological challenges posed by the modern intensive production system, the first feed offered to chicks already contains its natural microbial community, which could be enriched in pathogens during feed processing and transport. In addition to pathogens, the feed can also carry mycotoxins, heavy metals, pesticides, herbicides and other toxic contaminants. Many of these feed contaminants have aberrant effects on microbiota in adult birds, and those adverse effects would likely amplify in naïve birds. In other cases like radiation, chemical and toxic industrial waste, there was no literature on the effects on poultry intestinal microbiota, but it is safe to presume that they would not be beneficial.

The research in early gut colonisation in livestock and poultry is accelerating with the rise of modern high-throughput sequencing and other technologies, and more attention should be given to the precision designing of initial feed. In addition to the conventional starter, grower and finisher feed regimen, a carefully designed gut colonisation type of feed that would be offered immediately after arrival to the shed could be beneficial. Considering the speed of the gut colonisation process, it is possible that this could be offered only once as the first batch of feed. Enriching this first feed with probiotics and prebiotics could provide lifelong benefits. Different farming practices will complicate attempts to standardise new colonisation-targeted practices in poultry farms: time from hatch to placement on the shed floor, time spent in transport, type of transport, etc., vary significantly even between the sheds in the same farm, and more extremely on a global scale. Regardless of the challenges, research in this area to optimise the process will likely benefit bird welfare and productivity.

The present literature review implies the need for internationally regulated livestock feed testing due to the high import and export of livestock food products. From the gut microbiota establishment point of view, the feed offered to hatchlings during the critical first days of microbiota formation should be immaculate in terms of both biological and chemical contaminants and, if possible, enriched with beneficial and free of pathogenic bacteria, with nutritional composition highly supportive of fibre and other prebiotics loving bacteria.

## Data Availability

Not applicable.
